# Supplementary material for: TGF-β-dependent reprogramming of amino acid metabolism induces epithelial–mesenchymal transition in non-small cell lung cancers
Source: Commun Biol. 2021 Jun 24;4:782. doi: 10.1038/s42003-021-02323-7 (PMC8225889; doi:10.1038/s42003-021-02323-7)
Supplement: Supplementary file 2 — Supplementary Information [file 42003_2021_2323_MOESM2_ESM.pdf]

Supplementary Figures

TGF-β-dependent reprogramming of amino acid metabolism induces epithelial–mesenchymal transition in non-small cell lung cancers

Fumie Nakasuka[1,2,3,4,16], Sho Tabata[1,2,5,6,16,17]*, Takeharu Sakamoto[7,8], Akiyoshi Hirayama[1,2], Hiromichi Ebi[9], Tadaaki Yamada[10], Ko Umetsu[1], Maki Ohishi[1], Ayano Ueno[1], Hisatsugu Goto[11], Masahiro Sugimoto[1,2,12], Yasuhiko Nishioka[11], Yasuhiro Yamada[3,4], Masaru Tomita[1,2], Atsuo T. Sasaki[1,5,13,14], Seiji Yano[15], and Tomoyoshi Soga[1,2]

[1]Institute for Advanced Biosciences, Keio University, Tsuruoka, Yamagata 997-0052, Japan

[2]Systems Biology Program, Graduate School of Media and Governance, Keio University, Fujisawa, Kanagawa 252-0882, Japan

[3]Department of Computational Biology and Medical Sciences, Graduate School of Frontier Sciences, The University of Tokyo, Chiba 277-8561, Japan

[4]Division of Stem Cell Pathology, Center for Experimental Medicine and Systems Biology, Institute of Medical Science, The University of Tokyo, Tokyo 108-8639, Japan

[5]Division of Hematology and Oncology, Department of Internal Medicine, University of Cincinnati, Cincinnati, OH 45267, USA.

[6]Institute for Protein Research, Osaka University, Suita, Osaka 565-0871, Japan

[7]Division of Cellular and Molecular Biology, The Institute of Medical Science, The University of Tokyo, Minato-ku, Tokyo 108-8639, Japan

[8]Department of System Biology, Institute of Medical, Pharmaceutical and Health Sciences, Kanazawa University, Takaramachi, Kanazawa, Ishikawa 920-8640, Japan

[9]Division of Molecular Therapeutics, Aichi Cancer Center Research Institute, Nagoya, Aichi 464-8681, Japan.

[10]Department of Pulmonary Medicine, Kyoto Prefectural University of Medicine, Kamigyo-ku, Kyoto 602–8566, Japan

[11]Department of Respiratory Medicine and Rheumatology, Graduate School of Biomedical Sciences, Tokushima University, Tokushima, Tokushima 770-8503, Japan

[12]Health Promotion and Preemptive Medicine, Research and Development Center for Minimally Invasive Therapies, Tokyo Medical University, Shinjuku, Tokyo 160-0022, Japan

[13]Department of Cancer Biology, University of Cincinnati College of Medicine, OH 45267, USA

[14]Department of Neurosurgery, Brain Tumor Center at UC Gardner Neuroscience Institute, Cincinnati, OH 45267, USA

[15]Department of Medical Oncology, Kanazawa University Cancer Research Institute, Kanazawa University, Kanazawa, Ishikawa 920-0934, Japan
[16]These authors contributed equally to this work

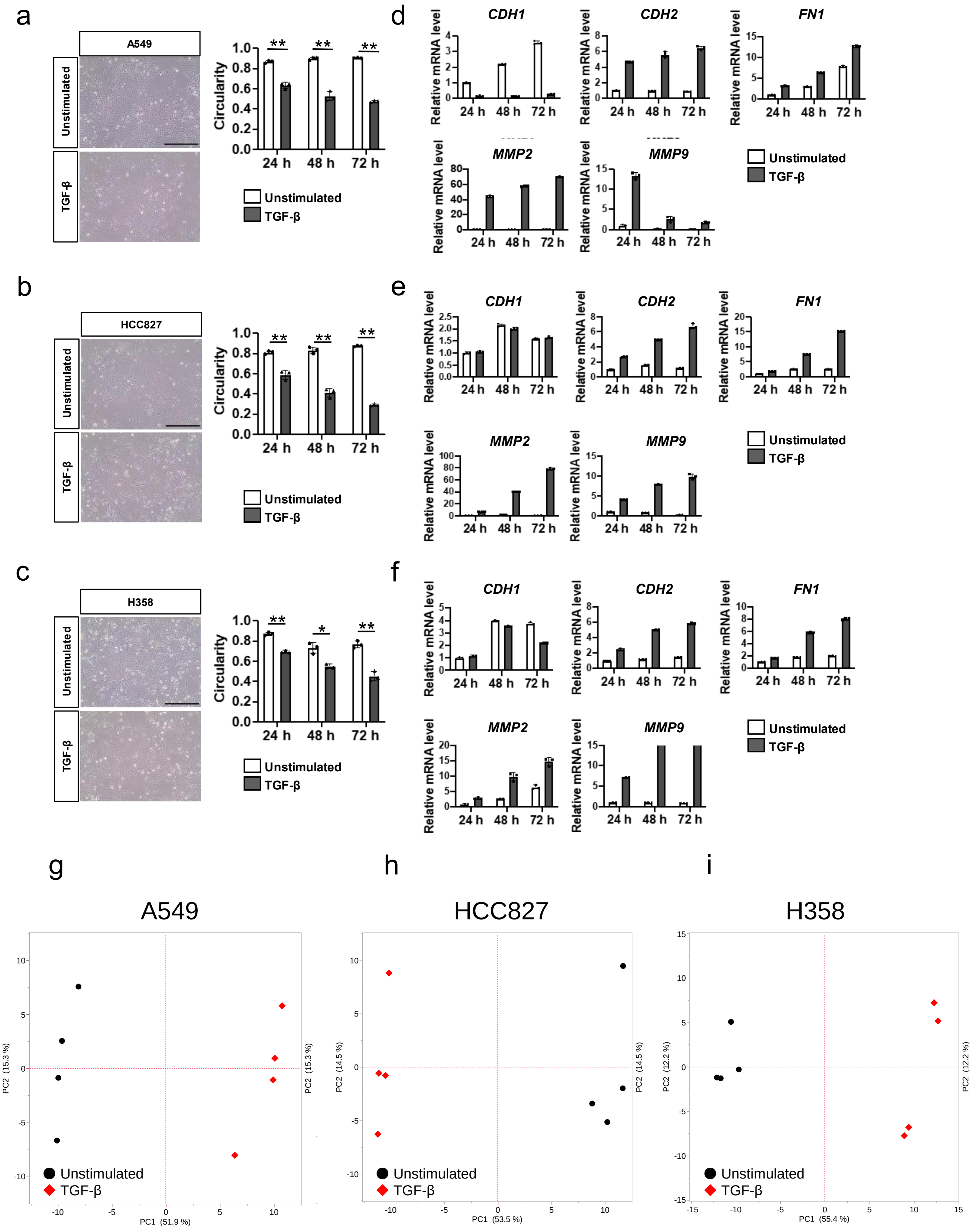
a
A549
Unstimulated
TGF-β
Circularity
24 h 48 h 72 h
Unstimulated
TGF-β
d
CDH1
CDH2
FN1
MMP2
MMP9
Relative mRNA level
Unstimulated
TGF-β
b
HCC827
c
H358
e
f
g
A549
h
HCC827
i
H358
PC1 (51.9 %)
PC2 (15.3 %)
PC1 (53.5 %)
PC2 (14.5 %)
PC1 (55.4 %)
PC2 (12.2 %)

j

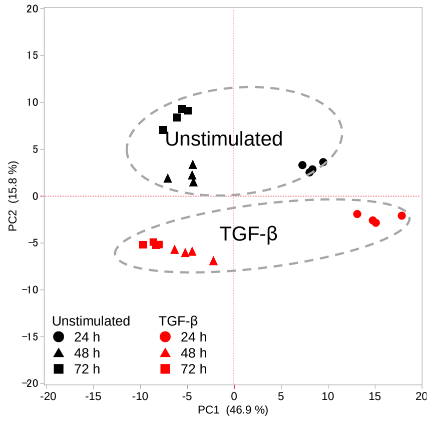
Unstimulated
TGF-β
PC2 (15.8 %)
PC1 (46.9 %)
Unstimulated
24 h
48 h
72 h
TGF-β
24 h
48 h
72 h

k

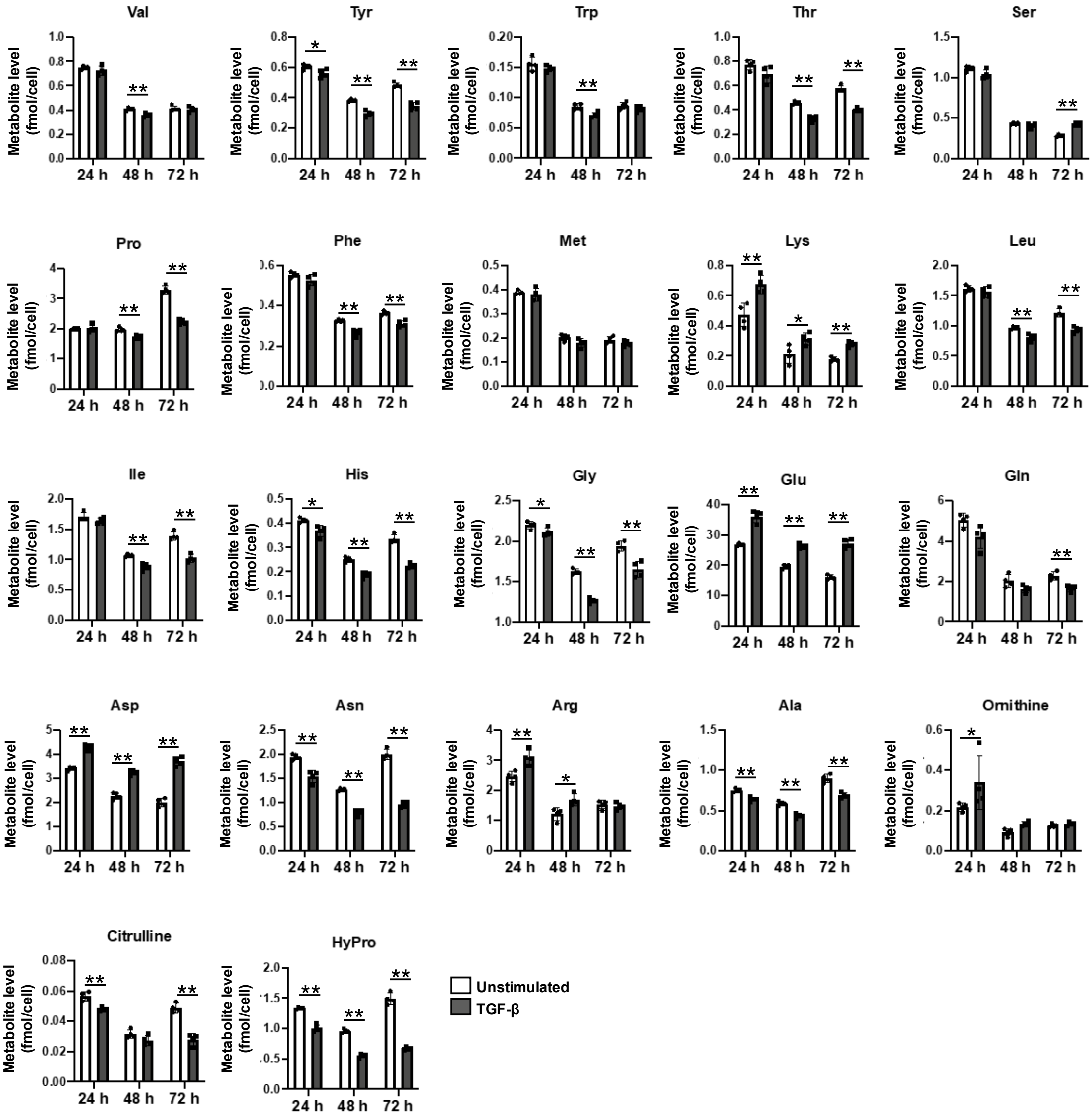
Val
Tyr
Trp
Thr
Ser
Pro
Phe
Met
Lys
Leu
Ile
His
Gly
Glu
Gln
Asp
Asn
Arg
Ala
Ornithine
Citrulline
HyPro
Metabolite level (fmol/cell)
24 h
48 h
72 h
Unstimulated
TGF-β

Supplementary Fig. 1 Related to Figure 1. (a–c) Morphological changes in TGF-β-stimulated (a) A549, (b) HCC827, and (c) H358 cells. Cells were seeded at a density of 2.0 × 10^5 cells/well in a 6-well plate and stimulated with 5 ng/mL TGF-β for 24, 48, and 72 h. Cells were observed by phase contrast inverted microscope at 100× magnification. Scale bar, 500 μm. Cell morphology was determined by calculating cell circularity using ImageJ software. (d–f) Expression of epithelial–mesenchymal transition (EMT) marker genes in (d) A549, (e) HCC827, and (f) H358 cells stimulated with or without 5 ng/mL TGF-β. mRNA expression levels of *CDH1*, *CDH2*, *FN1*, *MMP2*, and *MMP9* were measured using real-time PCR. (g–i) Score plots of principal component analysis (PCA) for metabolomics profiles of (g) A549, (h) HCC827, and (i) H358 cells stimulated with or without TGF-β (n = 4). Cells were cultured in the presence of 2 ng/mL TGF-β for 2–5 weeks to induce EMT. Metabolite levels were detected by capillary electrophoresis time-of-flight mass spectrometry (CE-TOFMS). The black and red plots indicate unstimulated and TGF-β-stimulated cells, respectively. (j) PCA for metabolomics profiles of A549 cells stimulated with 5 ng/mL TGF-β for 24, 48, and 72 h (n = 4). The black and red plots indicate unstimulated and TGF-β-stimulated cells, respectively. (k) Intracellular free-amino acids levels in A549 cells stimulated with TGF-β for 24, 48, and 72 h. The concentrations (fmol/cell) of each amino acid (n = 4). *$P < 0.05$, **$P < 0.01$.

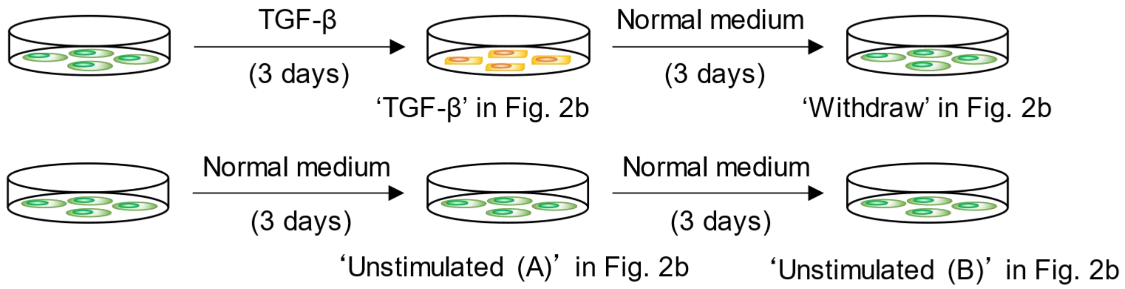

Supplementary Fig. 2 Related to Figure 2. Schematic diagram of sample preparation in Fig. 2b.

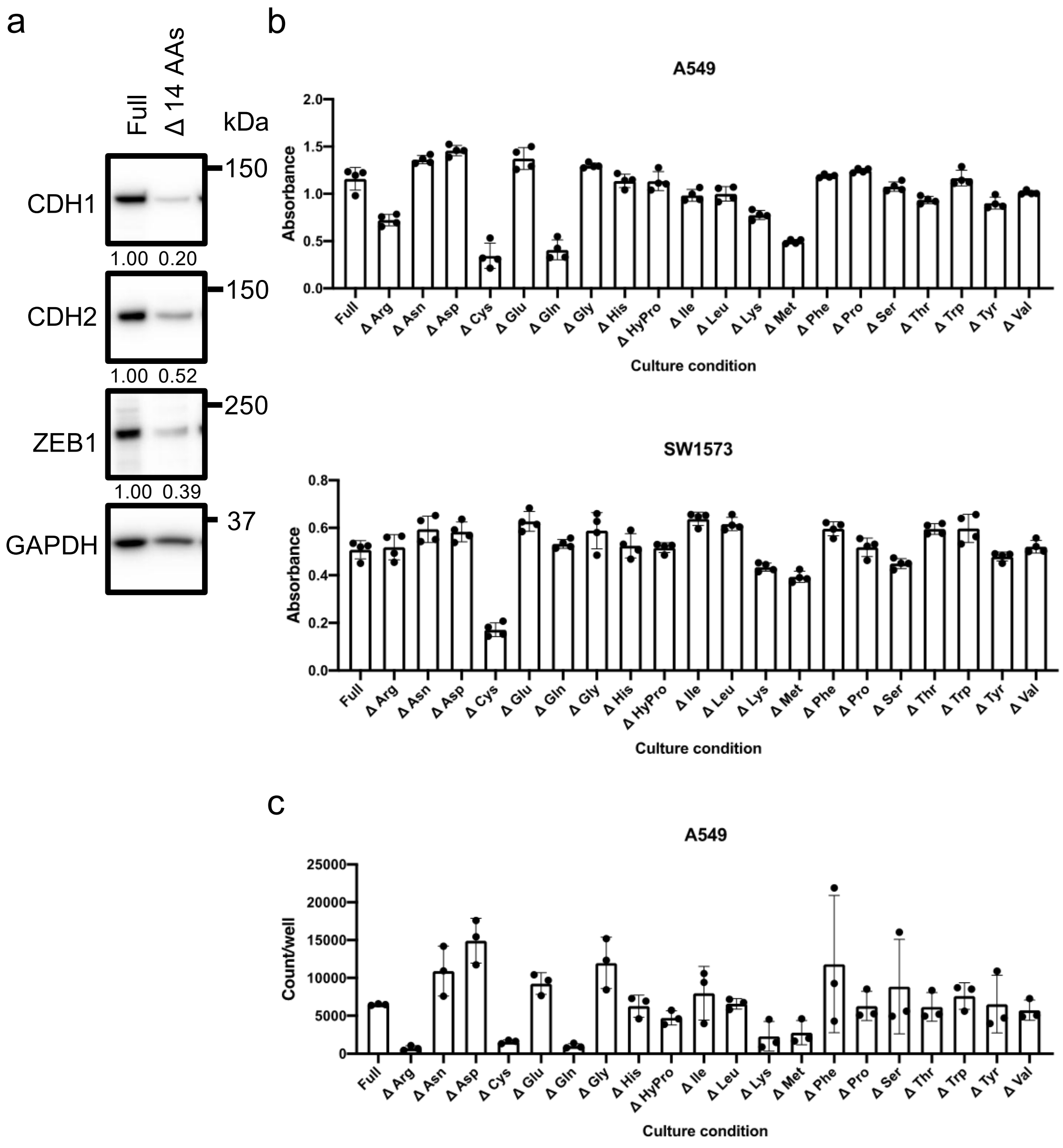

a
Full
Δ 14 AAs
kDa
CDH1
150
1.00 0.20
CDH2
150
1.00 0.52
ZEB1
250
1.00 0.39
GAPDH
37
b
A549
Absorbance
Culture condition
SW1573
Absorbance
Culture condition
Full
Δ Arg
Δ Asn
Δ Asp
Δ Cys
Δ Glu
Δ Gln
Δ Gly
Δ His
Δ HyPro
Δ Ile
Δ Leu
Δ Lys
Δ Met
Δ Phe
Δ Pro
Δ Ser
Δ Thr
Δ Trp
Δ Tyr
Δ Val
c
A549
Count/well
Culture condition

d

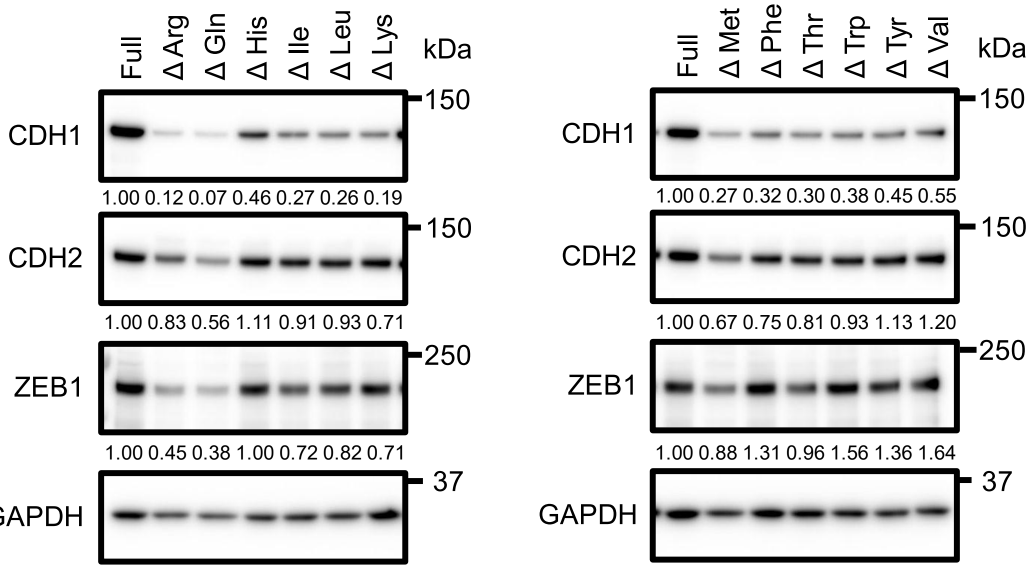
Full
Δ Arg
Δ Gln
Δ His
Δ Ile
Δ Leu
Δ Lys
kDa
CDH1
150
1.00 0.12 0.07 0.46 0.27 0.26 0.19
CDH2
150
1.00 0.83 0.56 1.11 0.91 0.93 0.71
ZEB1
250
1.00 0.45 0.38 1.00 0.72 0.82 0.71
GAPDH
37
Full
Δ Met
Δ Phe
Δ Thr
Δ Trp
Δ Tyr
Δ Val
kDa
CDH1
150
1.00 0.27 0.32 0.30 0.38 0.45 0.55
CDH2
150
1.00 0.67 0.75 0.81 0.93 1.13 1.20
ZEB1
250
1.00 0.88 1.31 0.96 1.56 1.36 1.64
GAPDH
37

e

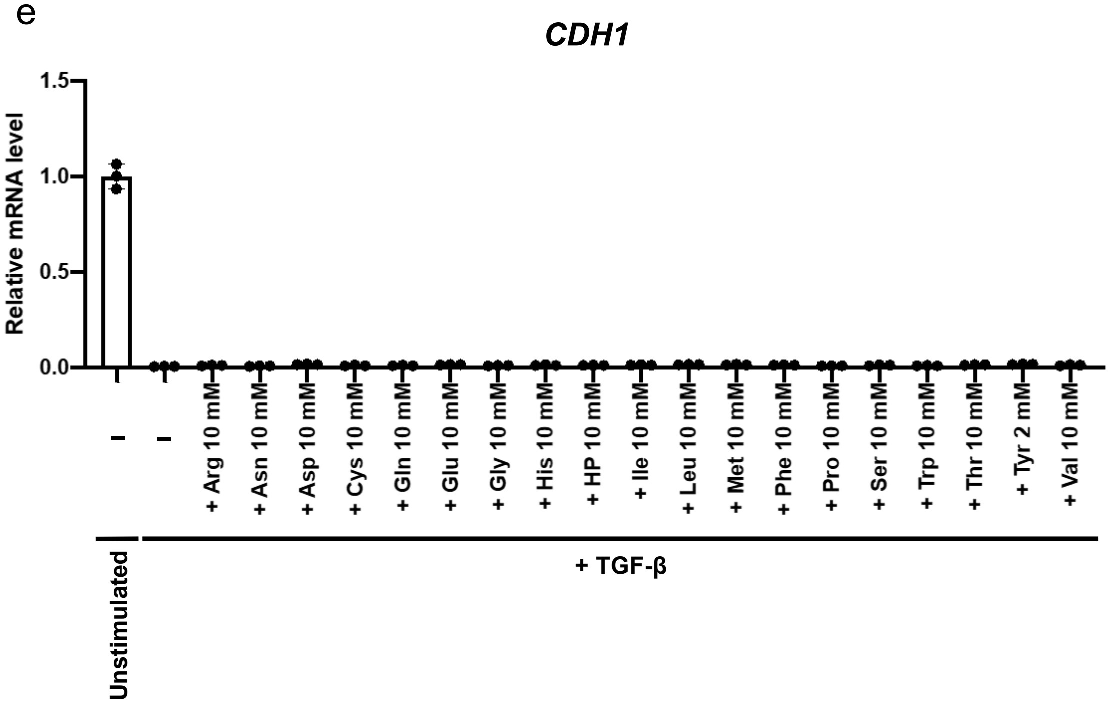
CDH1
Relative mRNA level
1.5
1.0
0.5
0.0
–
–
+ Arg 10 mM
+ Asn 10 mM
+ Asp 10 mM
+ Cys 10 mM
+ Gln 10 mM
+ Glu 10 mM
+ Gly 10 mM
+ His 10 mM
+ HP 10 mM
+ Ile 10 mM
+ Leu 10 mM
+ Met 10 mM
+ Phe 10 mM
+ Pro 10 mM
+ Ser 10 mM
+ Trp 10 mM
+ Thr 10 mM
+ Tyr 2 mM
+ Val 10 mM
Unstimulated
+ TGF-β

Supplementary Fig. 3 Related to Figure 3. (a) Effect of the combined deletion of Met, Val, Pro, Trp, Leu, Ile, Phe, Gly, Tyr, His, Ala, Thr, Asn, and hydroxyproline on the expression of EMT markers. CDH1, CDH2 and ZEB1 protein abundance quantified by western blotting using Image J software. Each protein is normalized to GAPDH levels and are presented as fold-change relative to those under regular conditions with full amino acids. (b) Effects of amino acid deprivation on cell viability in A549 and SW1573 cells assessed using MTT assay. (c) Effects of amino acid deprivation on cell viability in A549 cells assessed using DAPI staining. (d) Effects of amino acid depletion on CDH1, CDH2 and ZEB1 protein expression in A549 cells. (e) Effects of amino acid addition on mRNA expression of epithelial marker gene *CDH1* in A549 cells. Cells were treated with 5 ng/mL TGF-β and the indicated amino acid concentration for 72 h. mRNA expression levels were measured using real-time PCR. Data are presented as mean ± SD.

a

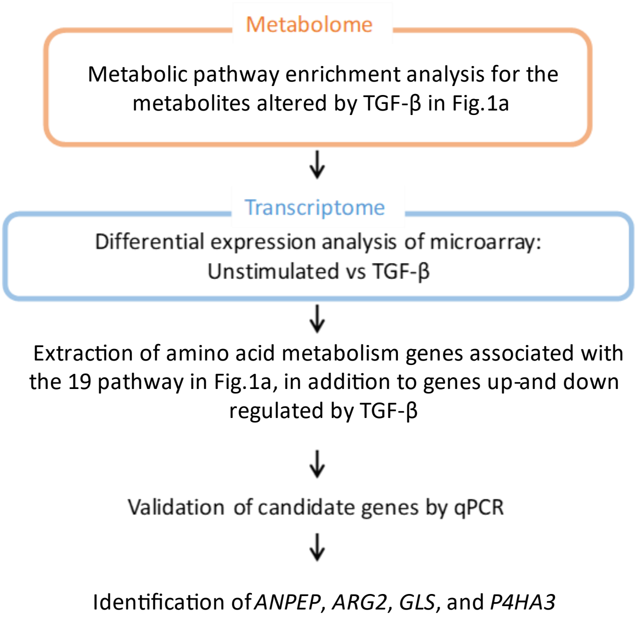

b

**Metabolic pathways**	Metabolism-related genes (F.C.)
Histidine-glutamate-glutamine metabolism	*GLS* (3.47)
Histidine-glutamate-glutamine and proline metabolism/ Rodent version	*P4HA3* (9.01) , *GLS* (3.47)
Nociception_Pro-nociceptive action of Nociceptin in spinal cord at low doses	-
Glycine, serine, cysteine and threonine metabolism	-
Methionine-cysteine-glutamate metabolism	*DNMT3B* (2.66)
Aspartate and asparagine metabolism	-
Neurophysiological process_GABAergic neurotransmission	-
L-Alanine, L-Cysteine, and L-Methionine metabolism / Human version	-
Urea cycle	*ARG2* (3.81)
Neurophysiological process_Circadian rhythm	*GLS* (3.47)
Nitrogen metabolism	*GLS* (3.47)
Propionate metabolism p.2	*OXCT1* (4.19)
Glutathione metabolism / Rodent version	*ANPEP* (3.99)
Regulation of lipid metabolism_PPAR regulation of lipid metabolism	-
Taurine and hypotaurine metabolism	-
Gamma-aminobutyrate (GABA) biosynthesis and metabolism	-
Arginine metabolism/ Rodent version	*ARG2* (3.81)
CTP/UTP metabolism	*DPYD* (2.22) , *CTPS* (3.13)
(L)-Arginine metabolism	*ARG2* (3.81)
Glycine links	-
Acetyl-CoA links	-

c

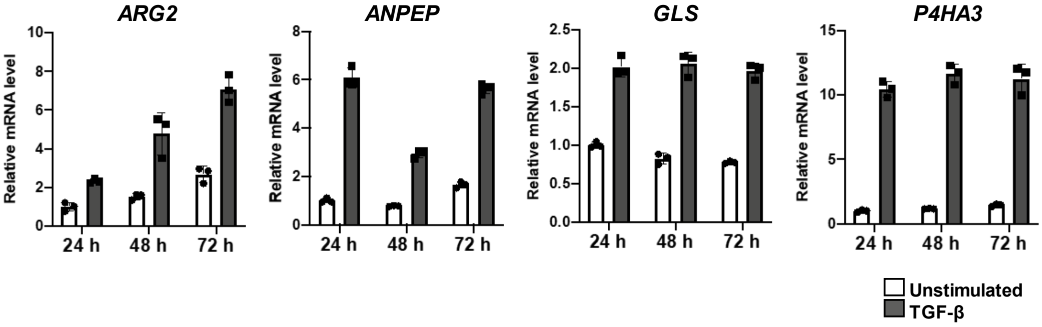
ARG2
ANPEP
GLS
P4HA3
Relative mRNA level
24 h
48 h
72 h
Unstimulated
TGF-β

d

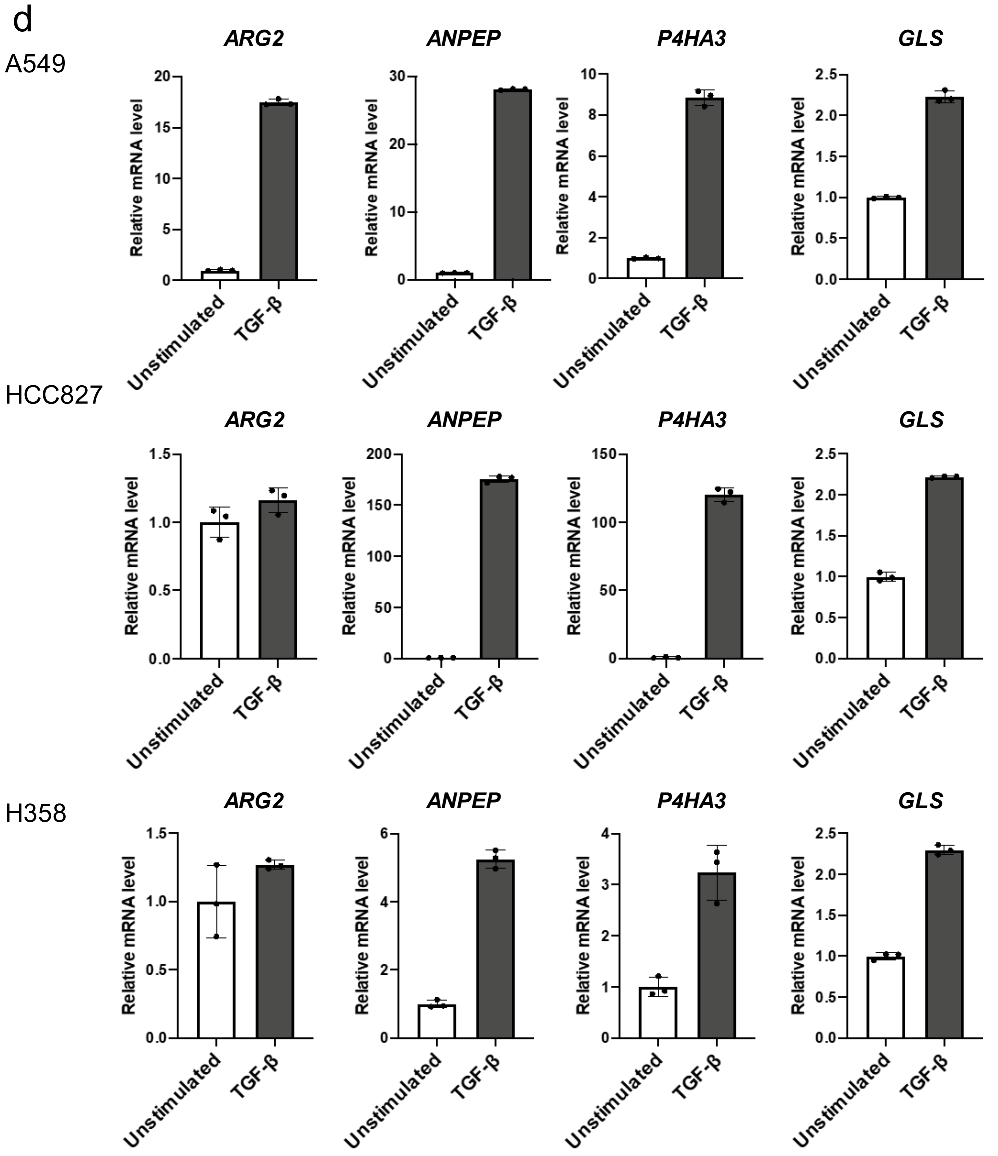
A549
HCC827
H358
ARG2
ANPEP
P4HA3
GLS
Relative mRNA level
Unstimulated
TGF-β

e

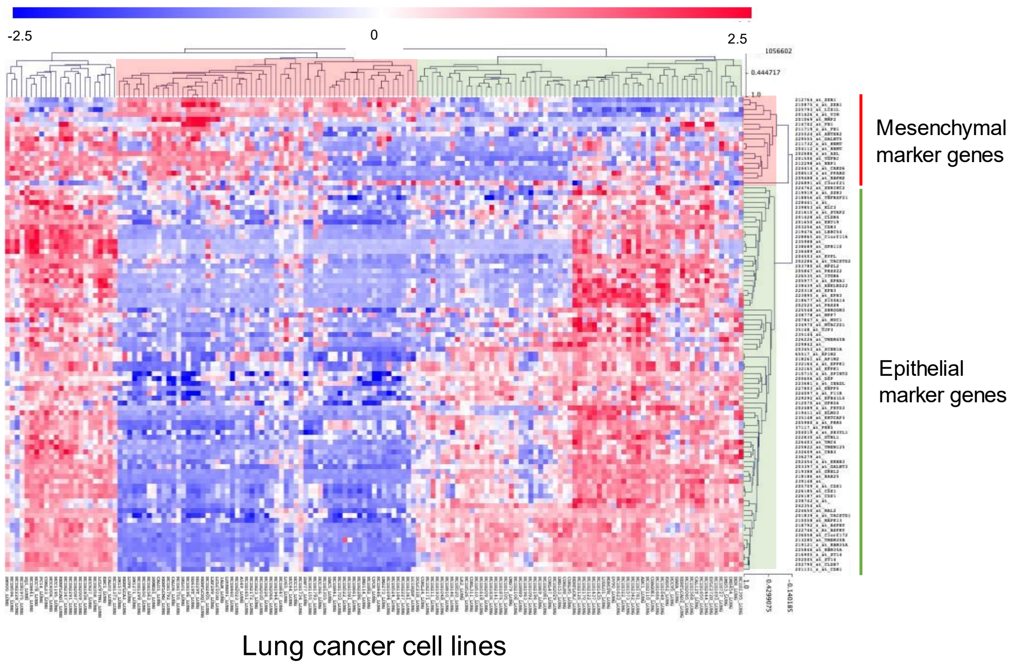

Supplementary Fig. 4 Related to Figure 4. (a) Scheme of data processing for identification of epithelial–mesenchymal transition (EMT)-related amino acid metabolism genes using datasets of metabolome and transcriptome. (b) List of 21 metabolic pathways mentioned in Fig. 1a and metabolism-related genes that were up- or down-regulated by TGF-β. mRNA levels of metabolism-related genes were measured by a microarray. (c) Expression of *ANPEP*, *ARG2*, *GLS*, and *P4HA3* in A549 cells stimulated with or without 5 ng/mL TGF-β for 24, 48, and 72 h. (d) Expression of *ANPEP*, *ARG2*, *GLS*, and *P4HA3* in A549, HCC827, and H358 cells. Cells were cultured in the presence of 2 ng/mL TGF-β for 2–5 weeks to induce EMT. mRNA levels were measured by real-time PCR. (e) Cluster analysis of 76 EMT marker genes in lung cancer cell lines from Cancer Cell Line Encyclopedia dataset.

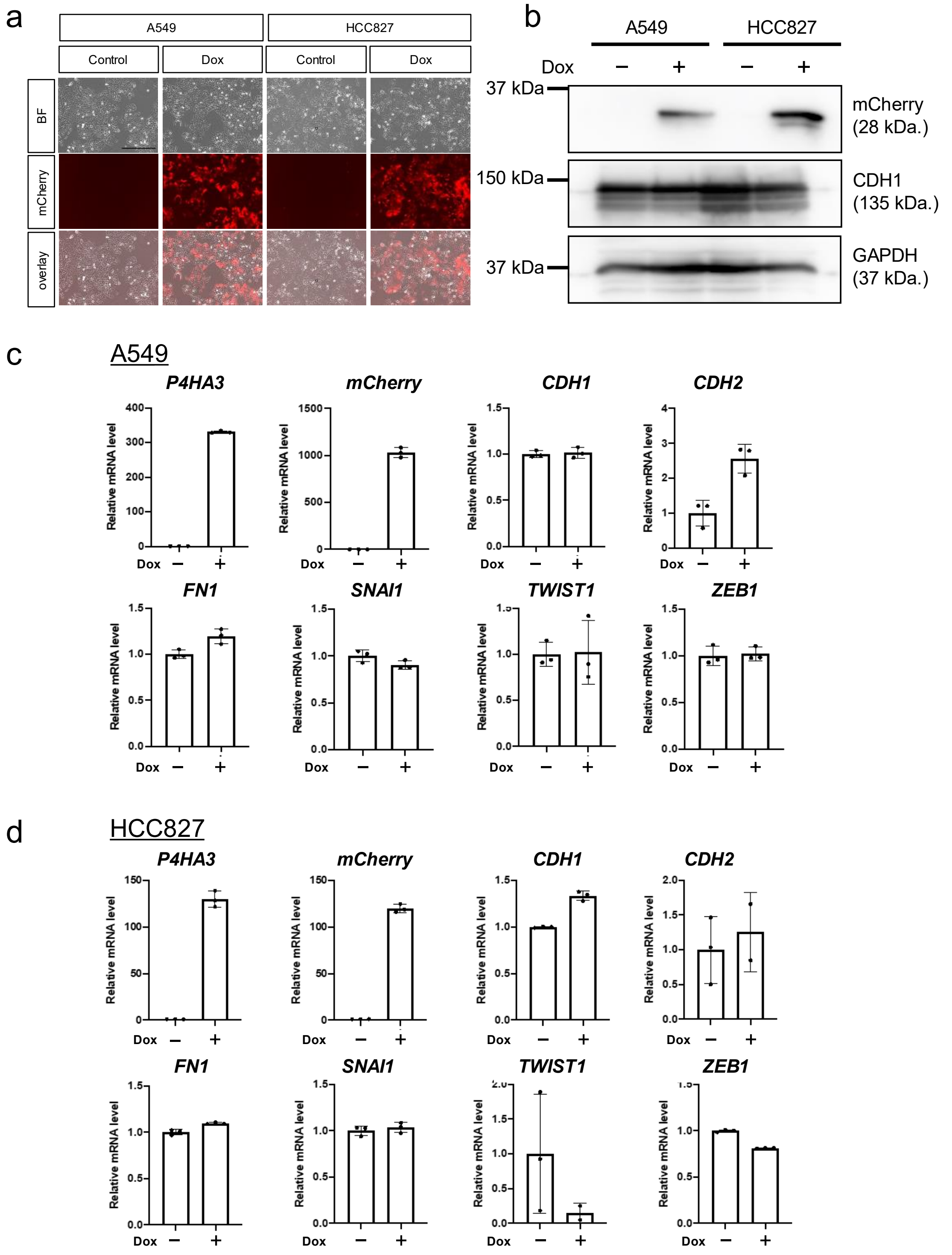
a
A549
HCC827
Control
Dox
Control
Dox
BF
mCherry
overlay
b
A549
HCC827
Dox − + − +
37 kDa
150 kDa
37 kDa
mCherry (28 kDa.)
CDH1 (135 kDa.)
GAPDH (37 kDa.)
c
A549
P4HA3
mCherry
CDH1
CDH2
FN1
SNAI1
TWIST1
ZEB1
Relative mRNA level
Dox − +
d
HCC827
P4HA3
mCherry
CDH1
CDH2
FN1
SNAI1
TWIST1
ZEB1
Relative mRNA level
Dox − +

e

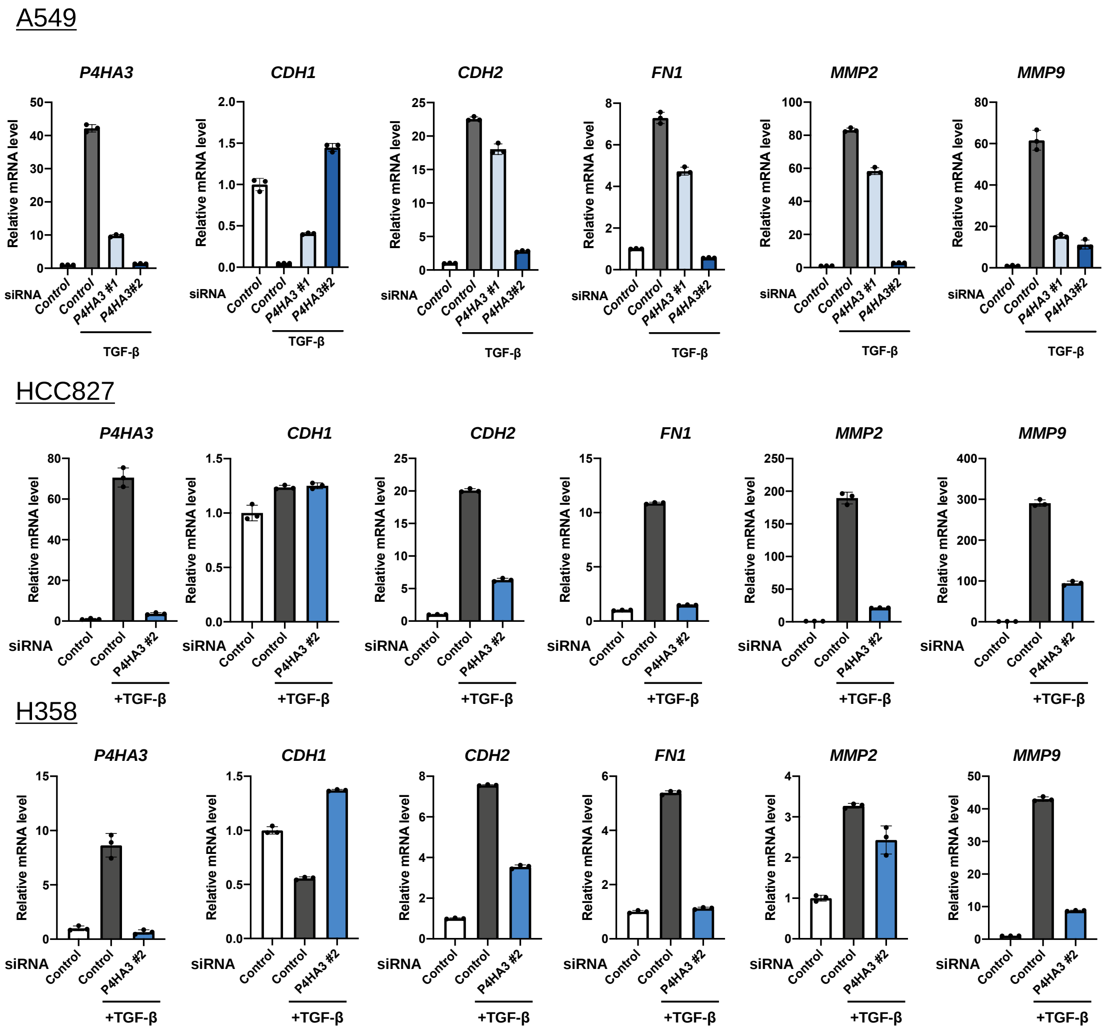

A549
P4HA3
CDH1
CDH2
FN1
MMP2
MMP9
Relative mRNA level
siRNA
Control
Control
P4HA3 #1
P4HA3#2
TGF-β
HCC827
H358
P4HA3 #2
+TGF-β

f

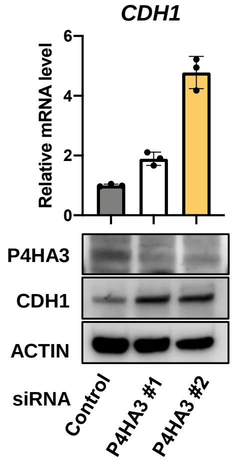

CDH1
Relative mRNA level
P4HA3
CDH1
ACTIN
siRNA
Control
P4HA3 #1
P4HA3 #2

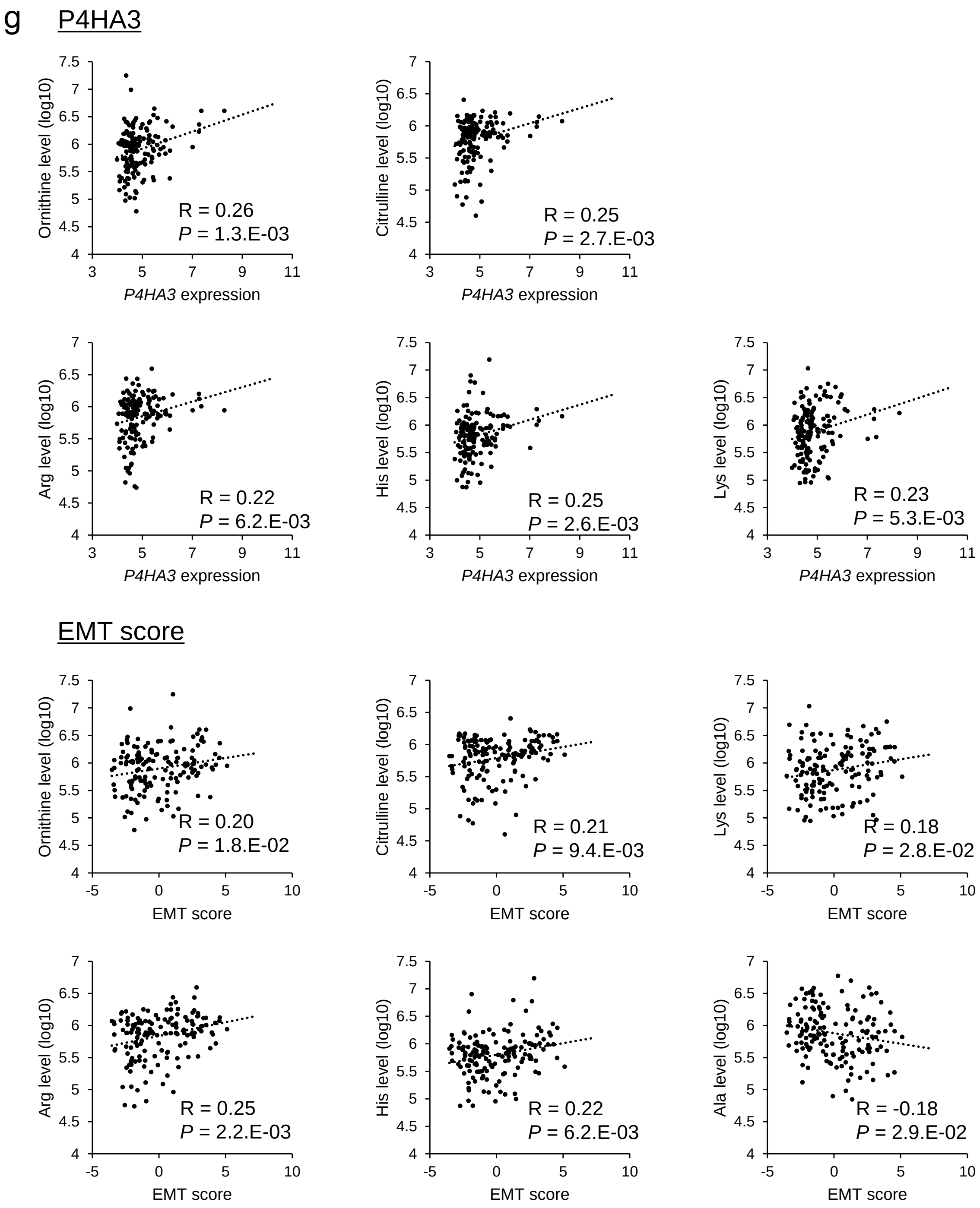
g
P4HA3
Ornithine level (log10)
R = 0.26
P = 1.3.E-03
P4HA3 expression
Citrulline level (log10)
R = 0.25
P = 2.7.E-03
P4HA3 expression
Arg level (log10)
R = 0.22
P = 6.2.E-03
P4HA3 expression
His level (log10)
R = 0.25
P = 2.6.E-03
P4HA3 expression
Lys level (log10)
R = 0.23
P = 5.3.E-03
P4HA3 expression
EMT score
Ornithine level (log10)
R = 0.20
P = 1.8.E-02
EMT score
Citrulline level (log10)
R = 0.21
P = 9.4.E-03
EMT score
Lys level (log10)
R = 0.18
P = 2.8.E-02
EMT score
Arg level (log10)
R = 0.25
P = 2.2.E-03
EMT score
His level (log10)
R = 0.22
P = 6.2.E-03
EMT score
Ala level (log10)
R = -0.18
P = 2.9.E-02
EMT score

Supplementary Fig. 5 Related to Figure 5. (a) Cell morphology and mCherry fluorescence in doxycycline (Dox) treated and untreated (Control) A549 and HCC827 cells observed by fluorescent microscope at 100× magnification. Scale bar, 500 µm. (b) Effect of P4HA3 overexpression on CDH1 protein levels in A549 and HCC827 cells as assessed by western blotting with GAPDH as the loading control. (c and d) Effect of P4HA3 overexpression on the expression of mRNA expression of various EMT markers in (c) A549 and (d) HCC827 cells measured by real-time PCR. Values are presented as mean ± SD from triplicate samples. (e) Effects of *P4HA3* siRNA on *EMT markers* and *P4HA3* mRNA expression in A549, HCC827, and H358 cells treated with TGF-β. (f) Effect of *P4HA3* siRNA on CDH1 levels in SW1573 cells. mRNA (upper) and protein (lower) levels of CDH1 were determined by real-time PCR and western blotting, respectively. Data for real-time PCR are presented as the mean ± SD. (g) Correlations between amino acids level, EMT score, and *P4HA3* mRNA expression in NSCLC cell lines from the CCLE dataset.

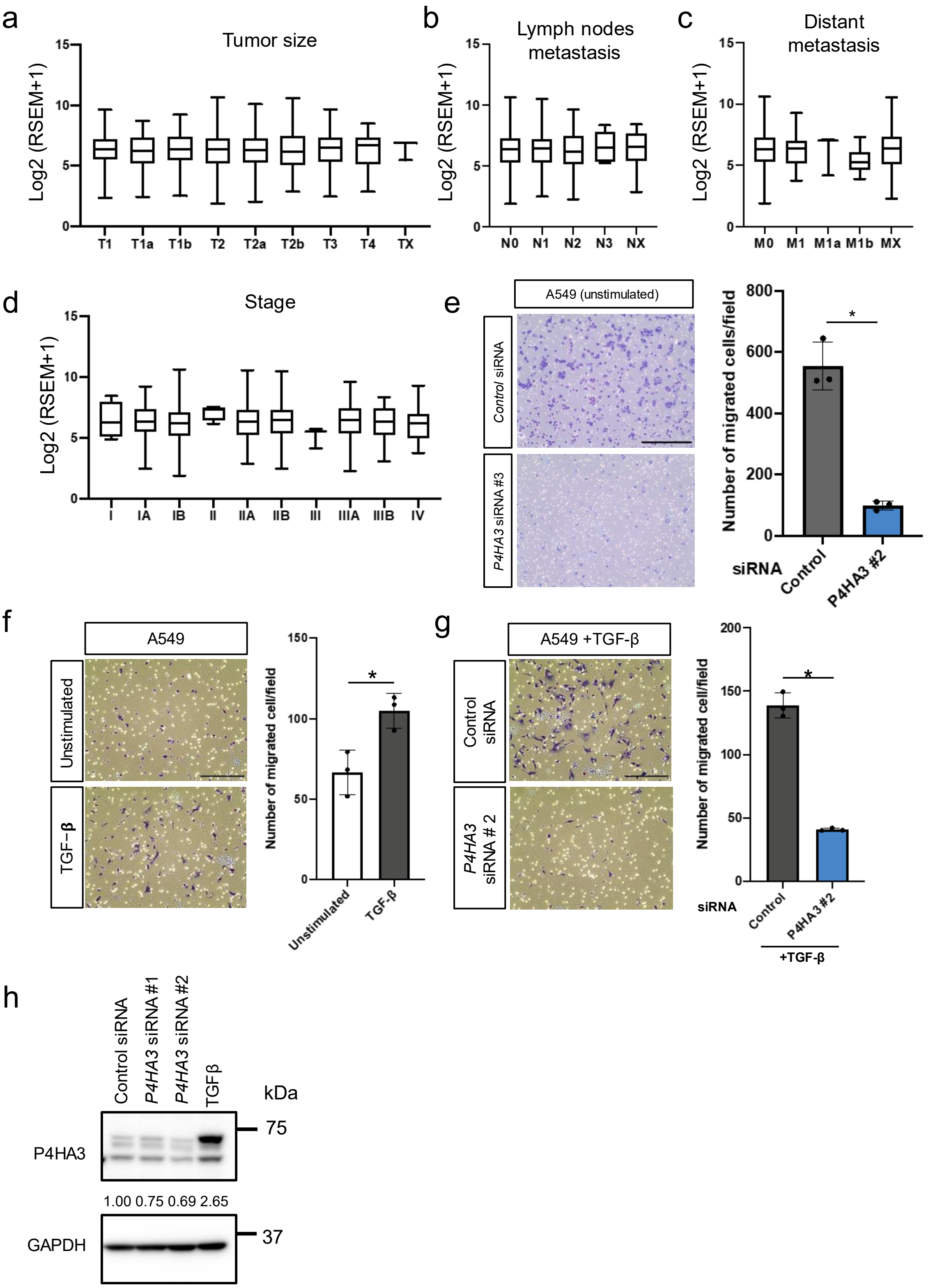
a
Tumor size
Log2 (RSEM+1)
15
10
5
0
T1
T1a
T1b
T2
T2a
T2b
T3
T4
TX
b
Lymph nodes metastasis
Log2 (RSEM+1)
N0
N1
N2
N3
NX
c
Distant metastasis
Log2 (RSEM+1)
M0
M1
M1a
M1b
MX
d
Stage
Log2 (RSEM+1)
I
IA
IB
II
IIA
IIB
III
IIIA
IIIB
IV
e
A549 (unstimulated)
Control siRNA
P4HA3 siRNA #3
Number of migrated cells/field
800
600
400
200
0
*
siRNA
Control
P4HA3 #2
f
A549
Unstimulated
TGF-β
Number of migrated cell/field
150
100
50
0
*
Unstimulated
TGF-β
g
A549 +TGF-β
Control siRNA
P4HA3 siRNA # 2
Number of migrated cell/field
200
150
100
50
0
*
siRNA
Control
P4HA3 #2
+TGF-β
h
Control siRNA
P4HA3 siRNA #1
P4HA3 siRNA #2
TGFβ
kDa
P4HA3
75
1.00 0.75 0.69 2.65
GAPDH
37

Supplementary Fig. 6 Related to Figure 6. (a–d) Box-and-whisker plot of *P4HA3* mRNA levels in (a) tumor size; T, (b) lymph node metastasis; N, (c) distant metastasis; M, and (d) stage. (e) Effect of *P4HA3* siRNA on migratory activity in A549 cells observed by bright field microscope at 100× magnification. Scale bar, 500 μm. Four random fields per sample were selected to enumerate the cells and each independent experiment was repeated in triplicate. Data are presented as migrated cells per field. *P < 0.01. (f) Migratory activity of A549 cells treated with TGF-β. Scale bar, 250 μm. (g) Effects of *P4HA3* siRNA on migratory activity of A549 cells treated with TGF-β. Scale bar, 250 μm. (h) Knockdown efficiency of *P4HA3* siRNA in A549 cells. Protein levels in each sample were determined by western blotting. GAPDH was the loading control.

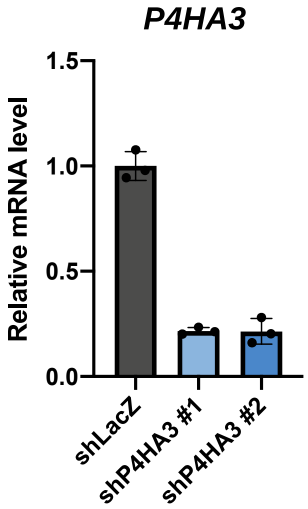

Supplementary Fig. 7 Related to Figure 7. Knockdown efficiency of *P4HA3* shRNA in A549 cells *in vitro*. mRNA levels in each sample were determined by real-time PCR. Values are presented as mean ± SD from triplicate samples.

Fig. 3b

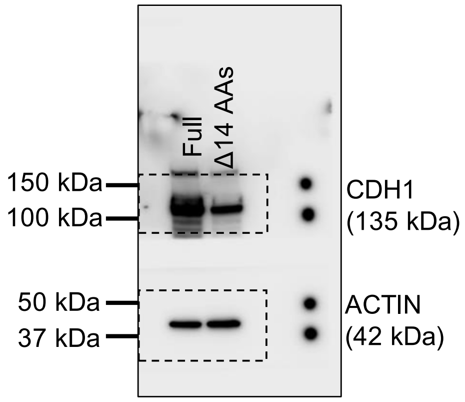

Fig. 3d: A549

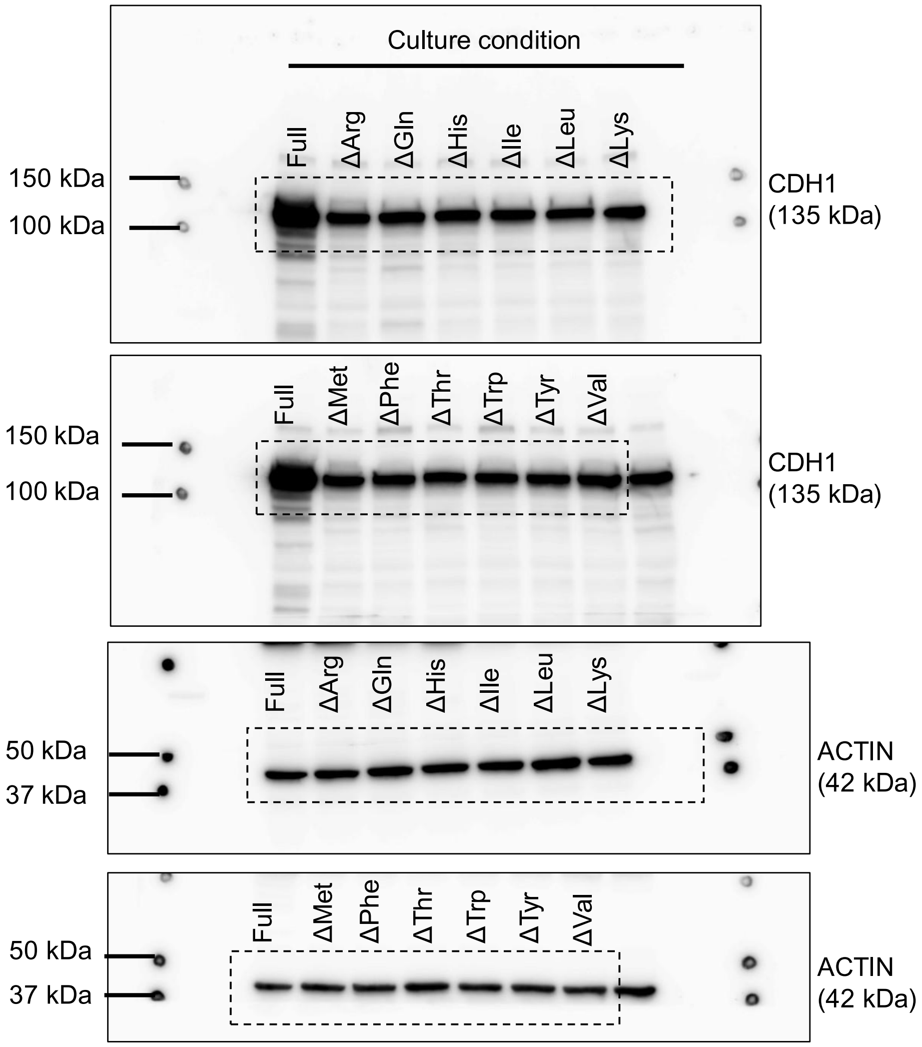

Fig. 3d: SW1573

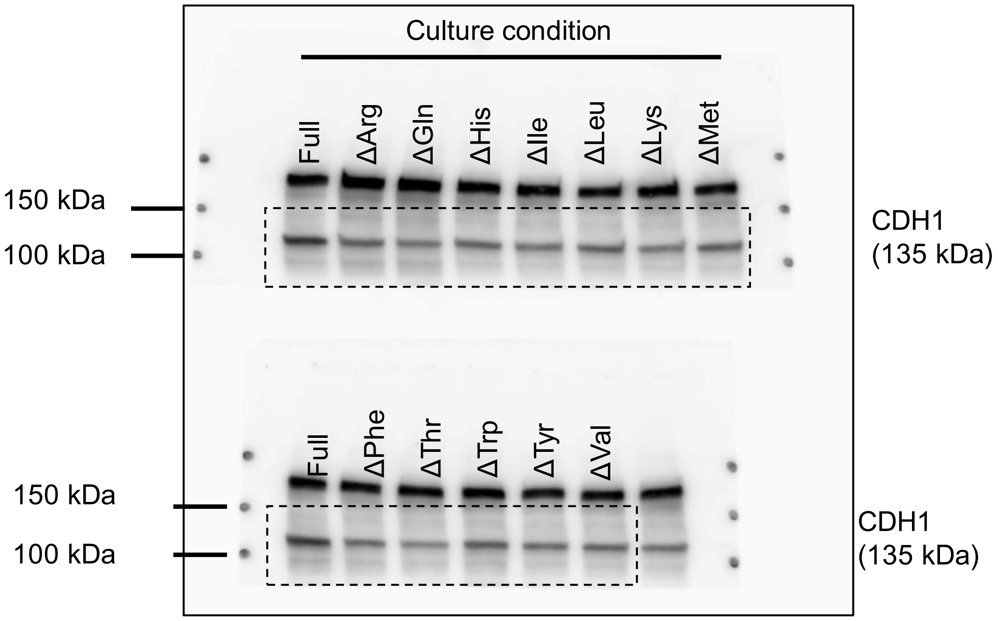

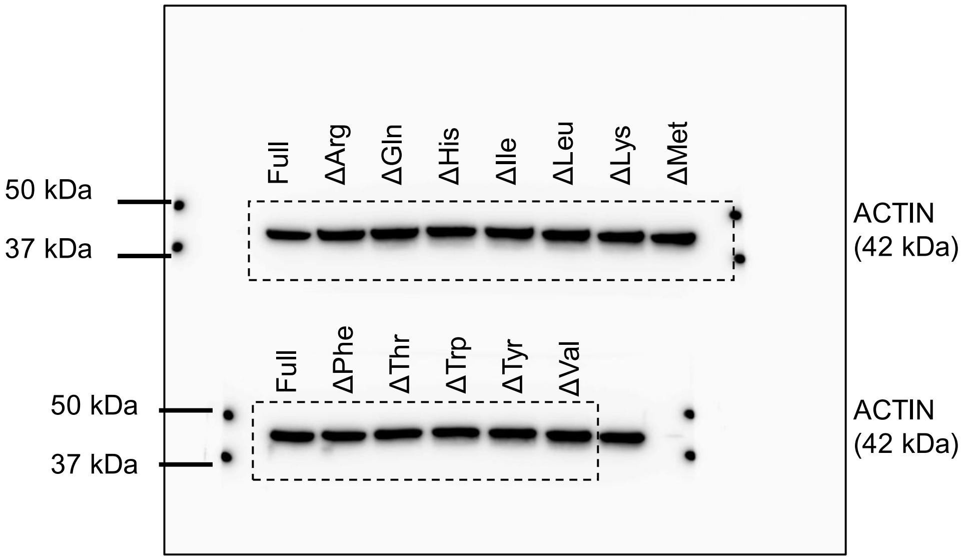

Supplementary Fig. 3a

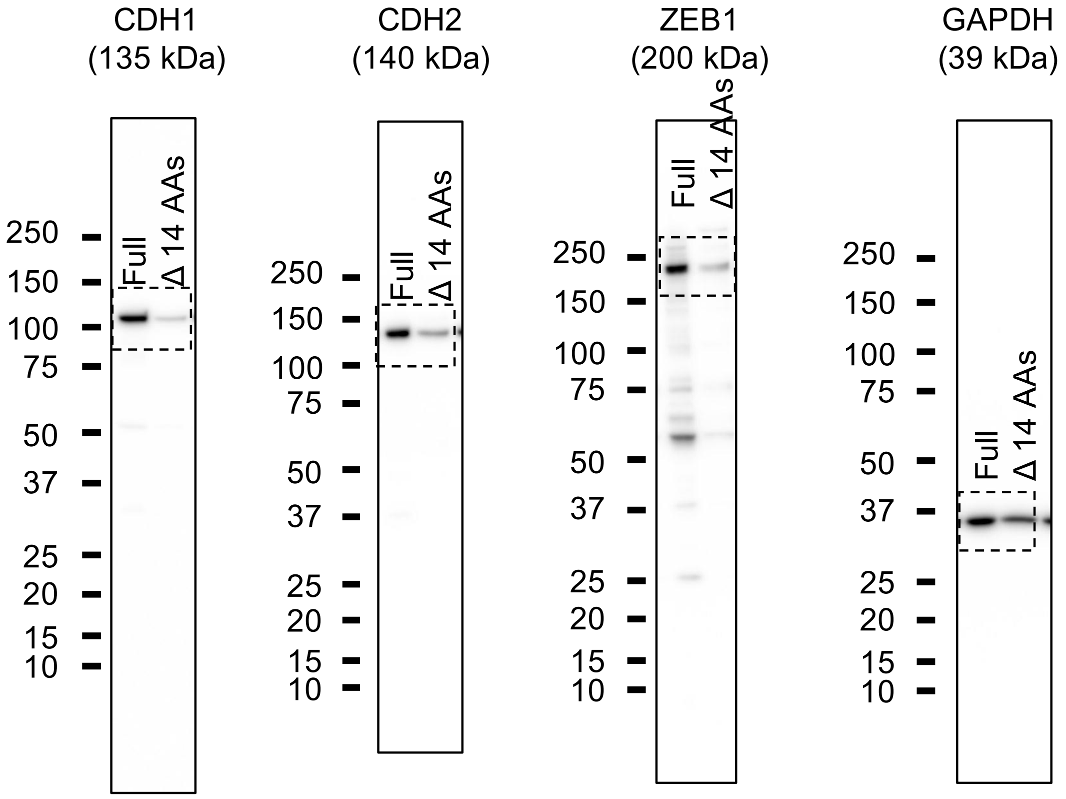

Supplementary Fig. 3d

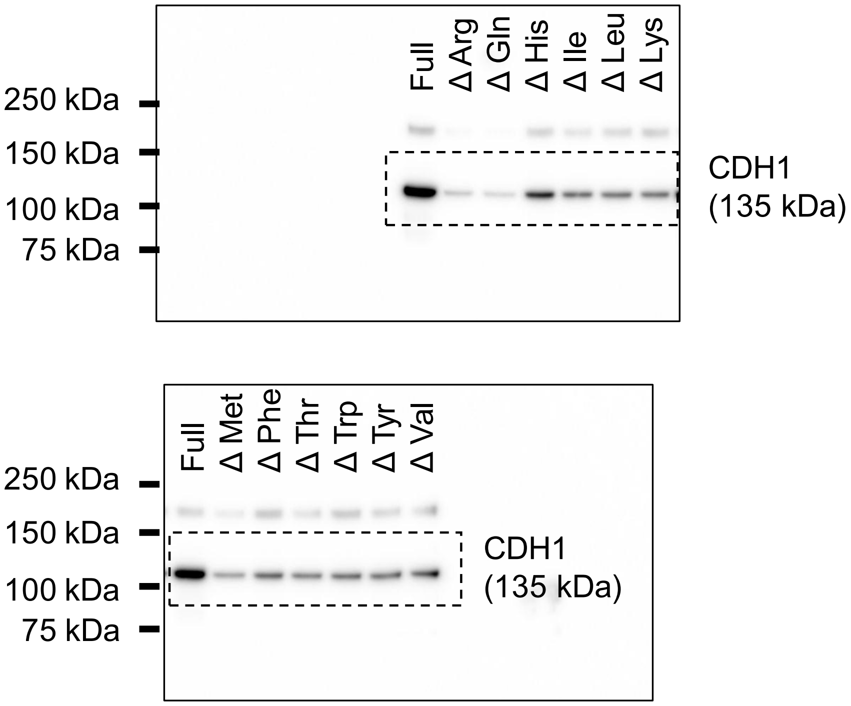

Supplementary Fig. 3d

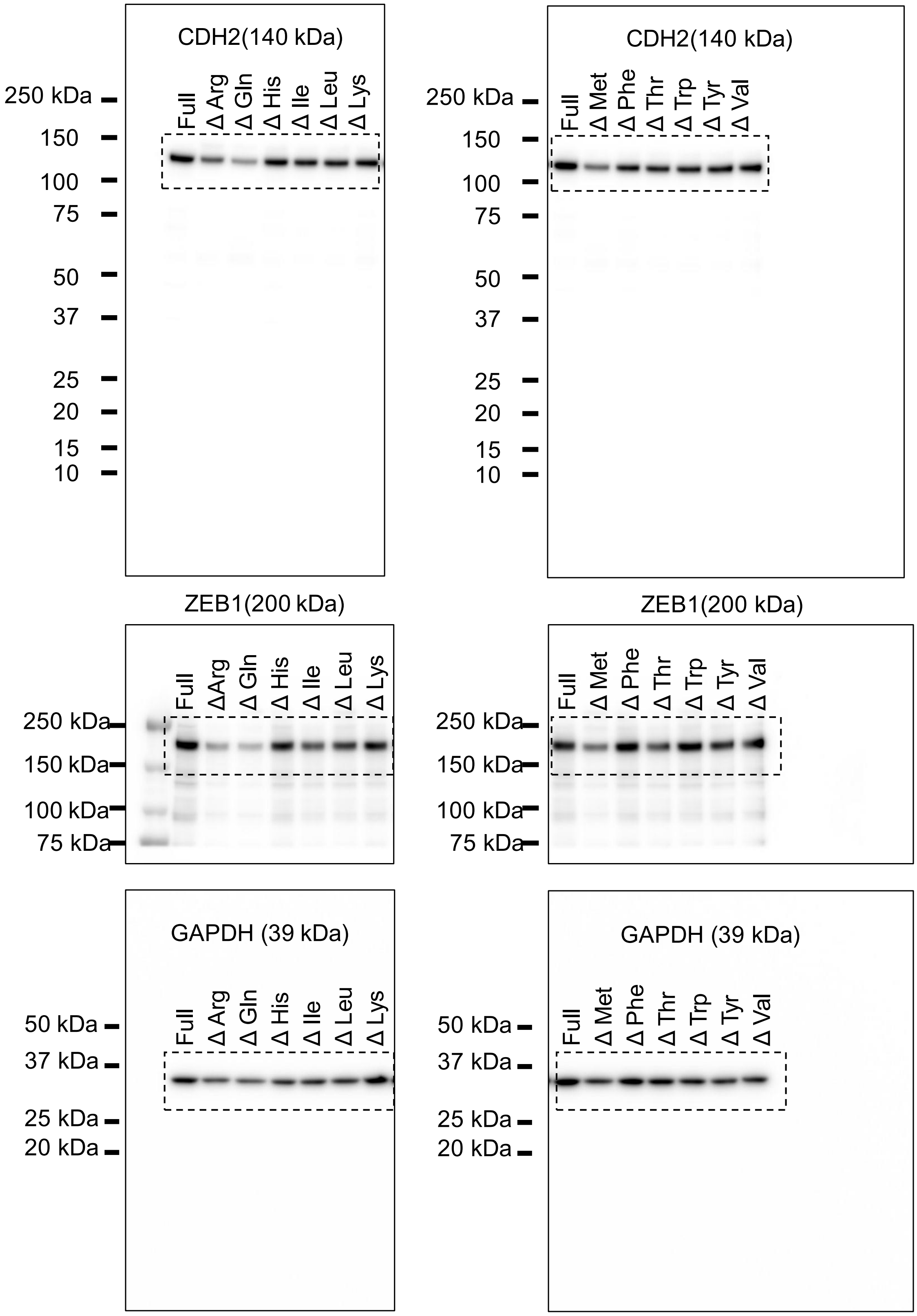

Fig. 4d

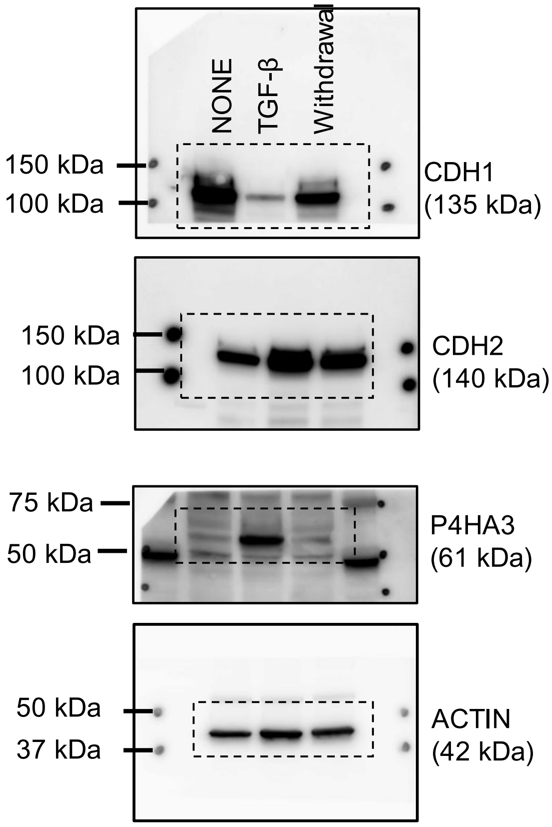

Supplementary Fig. 5b

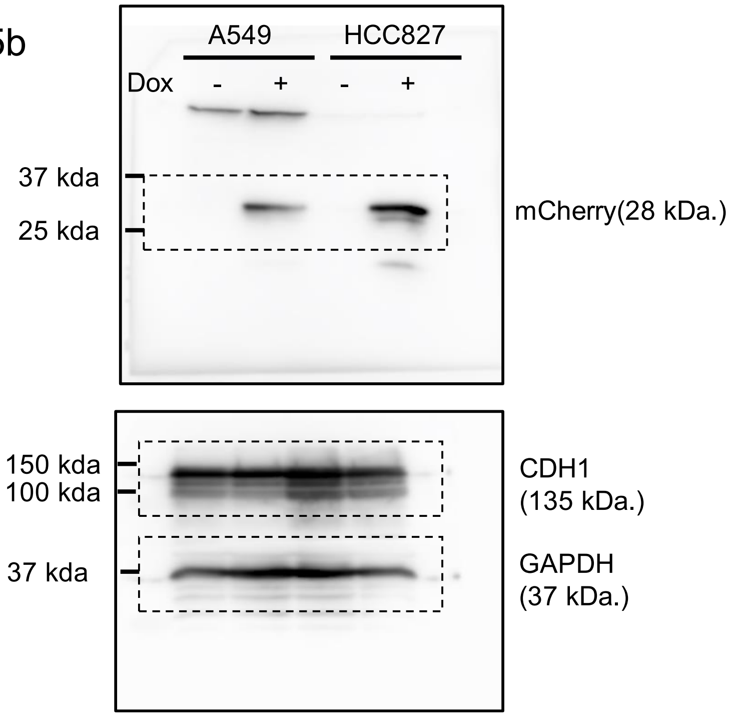

Supplementary Fig. 5f

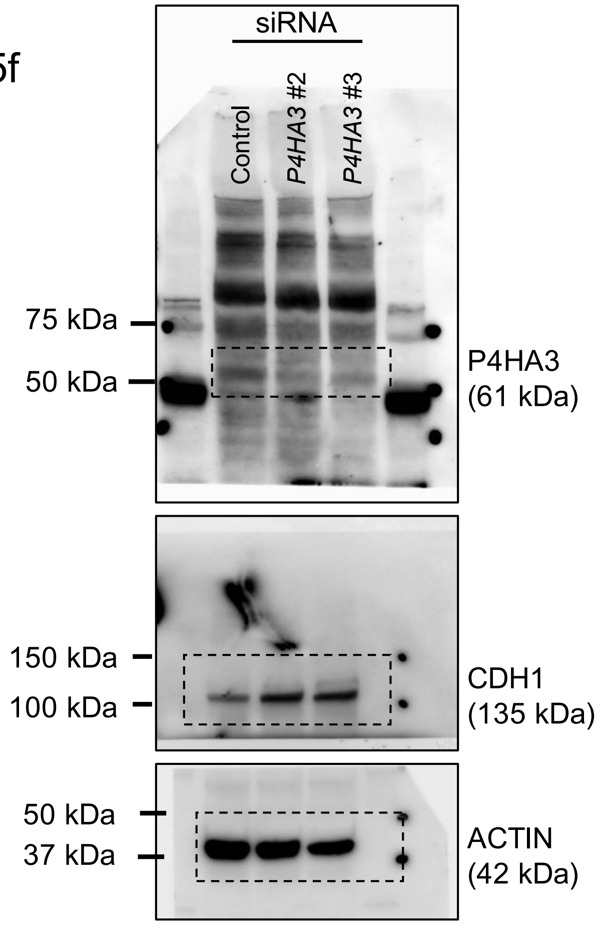

Supplementary Fig. 6h

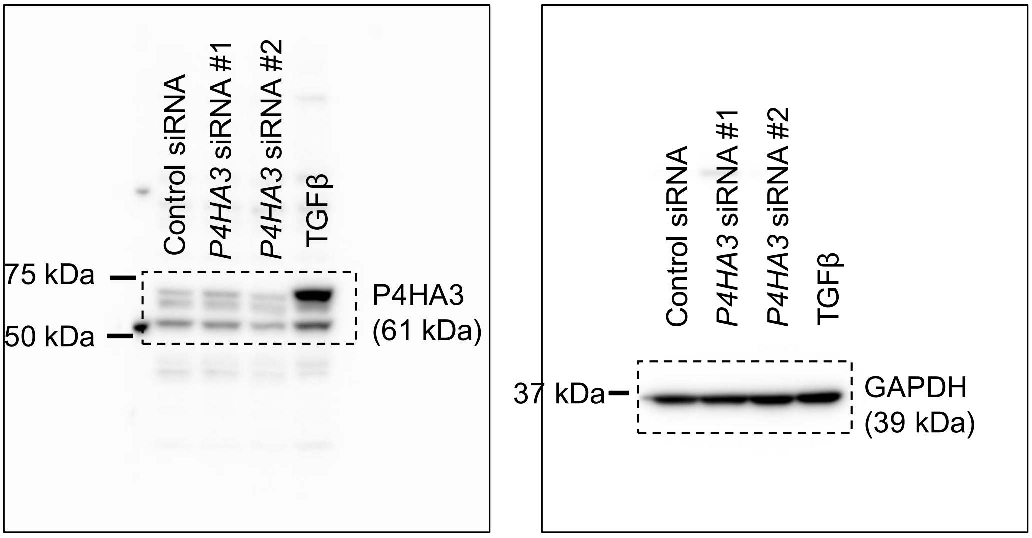

Supplementary Fig. 8 Uncropped images of western blots represented in Figs. 3b, 3d, 4d, and Supplementary Figs. 3a, 3d, 5b, 5f, 6h.
