# Supplementary material for: TGF-β-dependent reprogramming of amino acid metabolism induces epithelial–mesenchymal transition in non-small cell lung cancers
Source: Commun Biol. 2021 Jun 24;4:782. doi: 10.1038/s42003-021-02323-7 (PMC8225889; doi:10.1038/s42003-021-02323-7)
Supplement: Supplementary file 3 — Description of Additional Supplementary Files [file 42003_2021_2323_MOESM3_ESM.pdf]

Description of Additional Supplementary Files

File name: Supplementary Data 1
Description: Levels of metabolites in TGF-β-treated A549 cells.

File name: Supplementary Data 2
Description: Levels of metabolites in TGF-β-treated HCC827 cells.

File name: Supplementary Data 3
Description: Levels of metabolites in TGF-β-treated H358 cells.

File name: Supplementary Data 4
Description: Metabolic pathways that were altered by TGF-β in A549cells.

File name: Supplementary Data 5
Description: Metabolic pathways that were altered by TGF-β in HCC827 cells.

File name: Supplementary Data 6
Description: Metabolic pathways that were altered by TGF-β in H358 cells.

File name: Supplementary Data 7
Description: Levels of metabolites in A549 cells treated with TGF-β for 24, 48, and 72 h.

File name: Supplementary Data 8
Description: Levels of metabolites in the P4HA3-knockdown A549 cells stimulated with or without TGF-β.

File name: Supplementary Data 9
Description: Levels of metabolites in tumors derived from P4HA3-knockdown A549 cells.

File name: Supplementary Data 10
Description: List of amino acids.

File name: Supplementary Data 11
Description: Primer sequences.

File name: Supplementary Data 12

Description: Source data underlying the graphs.
