# Supplementary material for: TGF-β-dependent reprogramming of amino acid metabolism induces epithelial–mesenchymal transition in non-small cell lung cancers
Source: Commun Biol. 2021 Jun 24;4:782. doi: 10.1038/s42003-021-02323-7 (PMC8225889; doi:10.1038/s42003-021-02323-7)
Supplement: Supplementary file 16 — Reporting Summary [file 42003_2021_2323_MOESM16_ESM.pdf]

nature research

Corresponding author(s): Sho Tabata

Last updated by author(s): May 26, 2021

Reporting Summary

Nature Research wishes to improve the reproducibility of the work that we publish. This form provides structure for consistency and transparency in reporting. For further information on Nature Research policies, see our Editorial Policies and the Editorial Policy Checklist.

Statistics

For all statistical analyses, confirm that the following items are present in the figure legend, table legend, main text, or Methods section.

n/a	Confirmed	
[ ]	[x]	The exact sample size (*n*) for each experimental group/condition, given as a discrete number and unit of measurement
[ ]	[x]	A statement on whether measurements were taken from distinct samples or whether the same sample was measured repeatedly
[ ]	[x]	The statistical test(s) used AND whether they are one- or two-sided<br>*Only common tests should be described solely by name; describe more complex techniques in the Methods section.*
[ ]	[x]	A description of all covariates tested
[ ]	[x]	A description of any assumptions or corrections, such as tests of normality and adjustment for multiple comparisons
[ ]	[x]	A full description of the statistical parameters including central tendency (e.g. means) or other basic estimates (e.g. regression coefficient) AND variation (e.g. standard deviation) or associated estimates of uncertainty (e.g. confidence intervals)
[ ]	[x]	For null hypothesis testing, the test statistic (e.g. *F*, *t*, *r*) with confidence intervals, effect sizes, degrees of freedom and *P* value noted<br>*Give P values as exact values whenever suitable.*
[x]	[ ]	For Bayesian analysis, information on the choice of priors and Markov chain Monte Carlo settings
[x]	[ ]	For hierarchical and complex designs, identification of the appropriate level for tests and full reporting of outcomes
[ ]	[x]	Estimates of effect sizes (e.g. Cohen's *d*, Pearson's *r*), indicating how they were calculated

Our web collection on statistics for biologists contains articles on many of the points above.

Software and code

Policy information about availability of computer code

Data collection	Correlation analysis for amino acid level, EMT markers, and P4HA3 expression in lung cancer cell lines: Metabolome and transcriptome data for 187 lung cancer cell lines were obtained from the CCLE dataset (Barretina J. et al., Nature, 2012; Li H. et al., Nature Medicine, 2019).<br>Analysis of the association of P4HA3 with prognosis in patients with non-small cell lung cancer: The GSE datasets (GSE3141, GSE30219, and GSE31210) were used in the online survival analysis of KM Plotter.<br>The Cancer Genome Atlas data: Data for P4HA3 mRNA expression and TNM staging from "TCGA Lung Cancer (LUNG)" cohort were downloaded from UCSC Xena.
Data analysis	MultiExperiment Viewer (MeV) _4_8 ver.10.2<br>Microsoft excel 2016 for mac<br>GraphPad Prism v5.0 software<br>JMP 13<br>MasterHands Ver.2.17.3.18<br>BioVenn<br>KM-Plotter<br>R<br>UCSC Xena

For manuscripts utilizing custom algorithms or software that are central to the research but not yet described in published literature, software must be made available to editors and reviewers. We strongly encourage code deposition in a community repository (e.g. GitHub). See the Nature Research guidelines for submitting code & software for further information.

Data

Policy information about availability of data

All manuscripts must include a data availability statement. This statement should provide the following information, where applicable:

- Accession codes, unique identifiers, or web links for publicly available datasets
- A list of figures that have associated raw data
- A description of any restrictions on data availability

The data generated or analyzed during this study are provided in the article and supplementary files. Metabolome data are included in Supplementary Tables. Microarray data were deposited in the National Center for Biotechnology Information GEO with the accession code GSE136780. Source data are provided in Supplementary Data 1. Uncropped images of western blots are provided in Supplementary Fig. 5. All other data will be available upon reasonable request.

Field-specific reporting

Please select the one below that is the best fit for your research. If you are not sure, read the appropriate sections before making your selection.

- [x] Life sciences
- [ ] Behavioural & social sciences
- [ ] Ecological, evolutionary & environmental sciences

For a reference copy of the document with all sections, see nature.com/documents/nr-reporting-summary-flat.pdf

Life sciences study design

All studies must disclose on these points even when the disclosure is negative.

Sample size	Sample-size was calculated based on the similar study in the field.
Data exclusions	No data were excluded from the experiments and analysis.
Replication	Most experiments in vitro and cell-based assays were repeated in at least twice independent experiments, and the data were reproducible.
Randomization	In the experiment of xenograft, the mice were randomly allocated to the experimental groups.
Blinding	NA

Reporting for specific materials, systems and methods

We require information from authors about some types of materials, experimental systems and methods used in many studies. Here, indicate whether each material, system or method listed is relevant to your study. If you are not sure if a list item applies to your research, read the appropriate section before selecting a response.

Materials & experimental systems

n/a	Involved in the study	
[ ]	[x]	Antibodies
[ ]	[x]	Eukaryotic cell lines
[x]	[ ]	Palaeontology and archaeology
[ ]	[x]	Animals and other organisms
[x]	[ ]	Human research participants
[x]	[ ]	Clinical data
[x]	[ ]	Dual use research of concern

Methods

n/a	Involved in the study	
[x]	[ ]	ChIP-seq
[x]	[ ]	Flow cytometry
[x]	[ ]	MRI-based neuroimaging

Antibodies

Antibodies used	Mouse monoclonal anti-ACTIN: Santa Cruz Biotechnology, Inc.(Cat#sc-47778)<br>Rabbit polyclonal anti-P4HA3: Proteintech (Cat#23185-1-AP)<br>Rabbit monoclonal anti-CDH1: Cell Signaling Technology (Cat# 3195)<br>Mouse monoclonal anti-CDH2: Cell Signaling Technology (Cat# 14215)<br>Mouse monoclonal GAPDH: Ambion (Cat# AM4300)
Validation	All antibodies were validated by the manufacturers.

Eukaryotic cell lines

Policy information about cell lines

Cell line source(s)	A549, HCC827, H358, and SW1573 were obtained from the American Type Culture Collection (ATCC).
Authentication	Cell lines have been authenticated by ATCC.
Mycoplasma contamination	Cell lines were not regularly tested for mycoplasma contamination.
Commonly misidentified lines (See ICLAC register)	NA

Animals and other organisms

Policy information about studies involving animals; ARRIVE guidelines recommended for reporting animal research

Laboratory animals	6-week-old female BALB/c nude mice (Clea Japan, Tokyo, Japan)
Wild animals	NA
Field-collected samples	NA
Ethics oversight	This study was approved by the Animal Care and Use Committees of The Institute of Medical Science, The University of Tokyo.

Note that full information on the approval of the study protocol must also be provided in the manuscript.
